# LncRNA PCED1B-AS1 knockdown inhibits osteosarcoma via methylation-mediated miR-10a downregulation

**DOI:** 10.1186/s13018-022-03284-1

**Published:** 2022-10-23

**Authors:** Bing Wang, Li Yao, Yuefu Dong, Jian Liu, Jian Wu

**Affiliations:** Joint Surgery Department, The First People’s Hospital of Lianyungang, No.6 Zhenhua East Road, Lianyungang City, 222061 Jiangsu Province People’s Republic of China

**Keywords:** PCED1B-AS1, Osteosarcoma, miR-10a, Methylation, Proliferation

## Abstract

**Background:**

LncRNA PCED1B-AS1 (PCED1B-AS1) promotes glioma. This study aimed to investigate its role in osteosarcoma (OS).

**Methods:**

The study included 60 OS patients. Accumulation of miR-10a and PCED1B-AS1 in tissues from OS patients and cell lines was determined by RT-qPCR. Cell transfections were performed for interaction analysis. Participation of PCED1B-AS1 siRNA silencing and miR-10a overexpression in proliferation, invasion, and migration of U2OS and MG-63 cells was analyzed by cell proliferation assay and Transwell assay.

**Results:**

PCED1B-AS1 level was increased in OS and positively correlated with miR-10a level. In OS cells, PCED1B-AS1 siRNA silencing downregulated miR-10a. Methylation-specific PCR analysis showed that PCED1B-AS1 siRNA silencing decreased the methylation of miR-10a gene promoter. Moreover, PCED1B-AS1 siRNA silencing suppressed OS cell proliferation, invasion, and migration. In addition, miR-10a overexpression attenuated the effects of PCED1B-AS1 siRNA silencing.

**Conclusion:**

PCED1B-AS1 knockdown may inhibit OS cell proliferation and movement by regulating miR-10 gene methylation.

**Supplementary Information:**

The online version contains supplementary material available at 10.1186/s13018-022-03284-1.

## Background

As a type of primary malignant tumor originated from the skeleton, osteosarcoma (OS) is characterized by the formation of osteoid tissues and immature bones [1]. OS mainly affects teenagers and young adults [2], causing lifetime negative influence. It is estimated that OS accounts for more than 2% of malignancies in children blow 14 years old and 3% of malignancies in teens of 15 to 19 years old [1, 2]. OS diagnosed at early stages can be cured in most cases by neoadjuvant chemotherapy followed by surgical resection [3, 4]. However, tumor metastasis to other important organs, such as the lung and brain, is common in OS patients [5]. Distant tumor metastasis in OS patients is closely correlated with poor prognosis [6]. With localized tumors, more than 76% of OS patients can survive 5 years, while only about 25% of OS patients with distant metastasis can survive 5 years [5, 6]. Therefore, early diagnosis is the key to the survival of OS patients. However, due to lack of sensitive biomarkers, early diagnosis of OS is unlikely to be improved in the near future [3]. Therefore, in-depth investigation of the molecular pathogenesis of OS is still needed to improve the diagnosis and treatment of OS.

Numerous previous studies on the molecular mechanism of OS have identified multiple molecular regulators in OS [7]. Functional characterization of these molecular players provides novel insights in OS management [8–10]. Non-coding RNAs (ncRNAs), such as microRNAs (miRNAs) and long ncRNAs (lncRNAs), are not involved in protein-coding but can play critical roles in human diseases, such as cancers, by directly or indirectly regulating gene expression [11, 12]. In fact, altered expression of ncRNAs, such as miRNAs, has shown promising potentials in the diagnosis and prognosis of cancers, and regulating the expression of miRNAs is an emerging novel therapeutic approach for cancer management [13]. Therefore, ncRNAs are the novel gold mine to identify potential targets for cancer targeted therapy [11, 12]. For instances, certain differentially expressed lncRNAs and miRNAs may be detected to diagnose cancers at early stages [11, 12]. Some lncRNAs with critical functions in regulating cancer cell behaviors may be regulated to suppress cancer progression [11, 12]. Moreover, lncRNAs may sponge miRNAs to suppress their activities and interact with other pathways, such as methylation pathways, to affect gene expression, thereby participating in cancers [11, 12]. It has been well established that miRNAs can affect DNA methylation by targeting methylation-related proteins and DNA methyltransferases to regulate the expression of lncRNAs [13]. More recently, lncRNAs are reported to affect m6A methylation, thereby regulating the expression of protein-coding genes and miRNAs [11, 12]. However, the function of most lncRNAs in cancers still has not been investigated, which limits their application in cancer diagnosis and treatment. LncRNA PCED1B-AS1 (PCED1B-AS1) promotes several types of cancers, such as glioma [14], pancreatic ductal adenocarcinoma [15], and hepatocellular carcinoma [16], while its role in other cancers is unknown. We performed preliminary deep sequencing analysis and observed the altered PCED1B-AS1 expression and its positive correlation with miR-10a (data not shown), a critical player in cancers [17]. In most cases, miR-10a promotes the development of different types of cancers via different cancer-related pathways. For instance, miRNA-10a is overexpressed in oral cancer and promotes glucose metabolism by upregulating GLUT1 to promote cancer cell proliferation [17]. Therefore, it is reasonable to hypothesize that PCED1B-AS1 may regulate cancer-related pathways through miR-10a to participate in OS. We then studied the crosstalk between PCED1B-AS1 and miR-10a in OS.

## Methods

### Tissue collection

The study included 60 OS patients (37 males and 23 females; 12 to 33 years; 23.2 ± 3.4 years) who admitted to The First People's Hospital of Lianyungang between July 2016 and July 2019. The inclusion criteria were (1) OS patients diagnosed for the first time and (2) no therapy was initiated. The exclusion criteria were (1) patients complicated with other severe diseases, (2) patients with blood relationship and (3) recurrent cases. During biopsy, OS tissues and paired adjacent noncancerous tissues (normal bone tissues within 5 cm around tumors) were collected from each patient and freshly stored in liquid nitrogen before use. All tissue samples were confirmed by histopathological biopsy. The Ethics Committee of the aforementioned hospital approved this study (Ethical Approval No. #8631). Informed consent was obtained. Characteristics of patients are shown in Additional file 1: Table S1.

### OS cells and cell culture

Human OS cell lines U2OS, MG-63, HOS, SJSA-1, and 143B (ATCC, USA) and normal osteoblast cells hFOB1.19 (ATCC, USA) were included in this study. MG-63, HOS, hFOB1.19 and 143B cell lines and SJSA-1 cells were cultured in EMEM (ATCC, USA) with 10% FBS and RPMI-1640 medium (ATCC, USA) with 10% FBS, respectively, at 37 °C (or at 33.5 °C for hFOB1.19 cells) in an incubator with 5% CO_2_ and 95% humidity.

### Cell transfection

PCED1B-AS1 siRNA (5′-AAGCGGUUCUCGUGCCUCAGU-3′), NC siRNA (Cat# SIC001, Sigma-Aldrich), miR-10a (5′-UACCCUGUAGAUCCGAAUUUGUG-3′) or NC miRNA (5′-GGUUCGUACGUACACUGUUCA-3′) was transfected into cells using Lipofectamine® 2000 (Invitrogen). In each transfection, 1 × 10^7^ cells in a 10-cm dish were transfected with 50 nM miRNA and/or siRNA. NC siRNA- or NC miRNA-transfected cells were NC cells. Untransfected cells were control (C) cells. Subsequent experiments were performed at 48 h of post-transfection.

### Methylation specific PCR (MSP)

Total DNAs were extracted from U2OS and MG-63 cells using Quick-DNA Kit (ZYMO RESEARCH) and converted. The converted DNA samples were used as templates to perform MSP using 2X Taq FroggaMix (FroggaBio, USA) to analyze the methylation status of miR-10a gene with primers 5′-TTATTATTGTGTGTTCGGAAAATC-3′ (forward) and 5′-GTAACGCGCCTAACTATTTAACA-3′ (reverse) for methylated DNAs and 5′-TGTTTATTATTGTGTGTTTGGAAAATT-3′ (forward) and 5′-TCATAACACACCTAACTATTTAACAA-3′ (reverse) for un-methylated DNAs. PCR product was 1601 bp (from position -101 to -1701). PCR conditions were 5 min at 95 °C followed by 35 cycles of 95 °C for 30 s, 55 °C for 28 s and 72 °C for 35 s and a final extension at 72 °C for 10 min. All PCRs were conducted on a Bio-Rad C1000 PCR machine (Bio-Rad).

### RNA preparation and RT-qPCR

After RNA preparation using Direct-zol RNA, DNase I-digested RNA samples were used to prepare cDNA samples. With cDNA samples as templates, qPCRs were performed with 18S rRNA internal control to measure the accumulation levels of both PCED1B-AS1 and miR-10a. The method of 2^−∆∆Ct^ was used for data normalizations. The following specific primers were employed: PCED1B-AS1 forward 5′-AAGGGGAAAGGAGGAAGTGAGAAG-3′ and reverse 5′-GGAAGCCAGTGAGCCAGGAGT-3′ and miR-10a forward 5′-CAGTGCAGGGTCCGAGGT-3′ and reverse 5′-GCCGTAC CCTGTAGATCCGAA-3′. PCR conditions were 1 min at 95 °C followed by 40 cycles of 95 °C for 10 s and 57 °C for 40 s. All qPCRs were conducted on BioRad CFX96 Touch Real Time PCR (Bio-Rad).

### Cell proliferation assay

The proliferation of both U2OS and MG-63 cells after transfections was analyzed using a CCK-8 kit (Dojindo). Briefly, cells were harvested and cultured at 37 °C in a 96-well plate with 3000 cells in 0.1 ml medium per well. Three replicate wells were set for each experiment. Cell culture was performed for 48 h, followed by adding CCK-8 solution to 10%. After incubation with CCK-8 for 4 h, OD values at 450 nm were measured.

### Transwell assay

Transwell Inserts (8.0 μm, Corning) were used and cells in 

non-serum medium were added to the upper chamber. To induce cell movement, FBS was added to 20% in the lower chamber, and the upper chamber was filled with 6000 cells in serum-free media. After incubation at 37 °C for 24 h, cells on the lower membranes were stained with 1% crystal violet (Sigma-Aldrich) and counted.

### Statistical analysis

Three independent replicates were included in each experiment, and mean ± SD values were used to express the data. Paired tissues (paired *t* test) and multiple groups (ANOVA Tukey’s test) were compared. The 60 OS patients were divided into high and low PCED1B-AS1 level groups (n = 30, cutoff value = median level of PCED1B-AS1 in OS tissues). Chi-squared test was applied to explore the associations between patients’ clinical characteristics and PCED1B-AS1 expression levels. Correlations were explored by performing Pearson’ correlation coefficient. *p* < 0.05 was statistically significant.

## Results

### PCED1B-AS1 and miR-10a expression was altered in OS

**Fig. 1:**
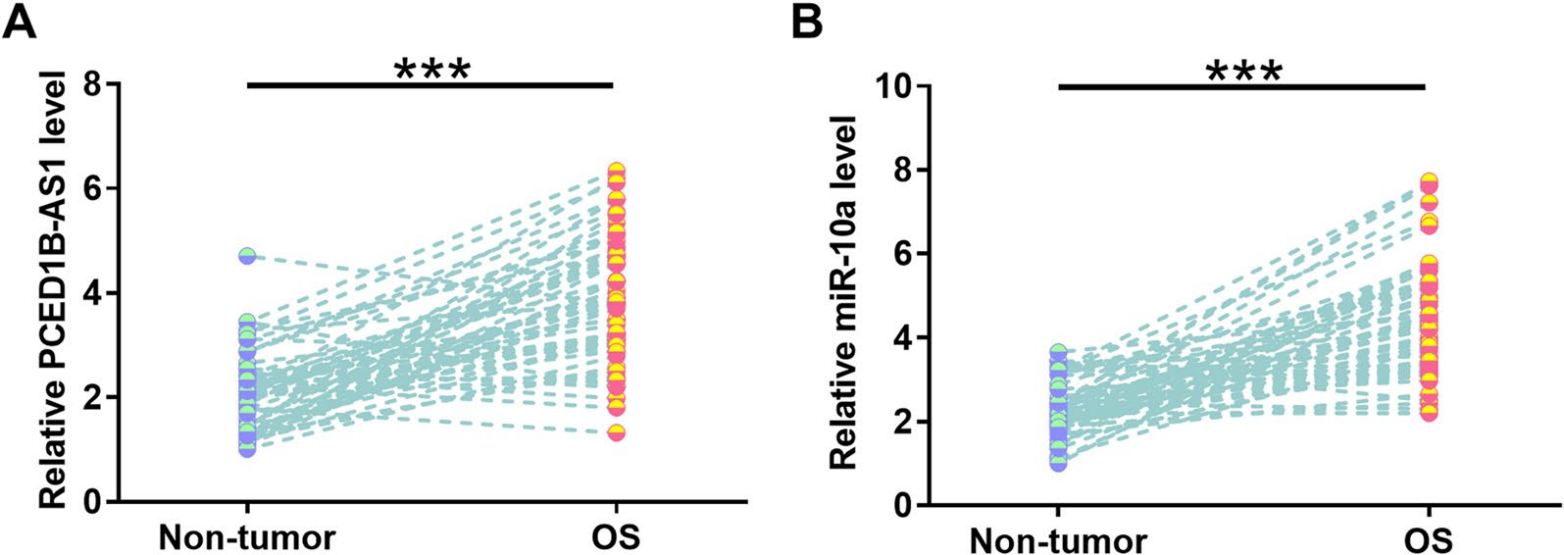
PCED1B-AS1 and miR-10a expression was altered in OS. Expression of PCED1B-AS1 (**A**) and miR-10a (**B**) in 60 paired tissues was determined by RT-qPCR. ***p < 0.001

PCED1B-AS1 level was increased by 1.79-fold was in OS tissues compared to non-tumor tissue samples (Fig. 1A, *p* < 0.001). Chi-squared test was applied to explore the associations between patients’ clinical characteristics and PCED1B-AS1 expression levels. It was observed that PCED1B-AS1 expression was correlated with TNM stage, tumor metastasis, tumor size, and Enneking staging of OS, but not other factors (Additional file 1: Table S1, *p* < 0.05), suggesting the potential involvement of PCED1B-AS1 in the progression of OS. In addition, RT-qPCR analysis revealed that miR-10a expression levels were increased by 1.86-fold in OS tissues in comparison with the non-tumor tissues (Fig. 1B, *p* < 0.001).

### Expression levels of PCED1B-AS1 and miR-10a were positively correlated across OS tissues

**Fig. 2:**
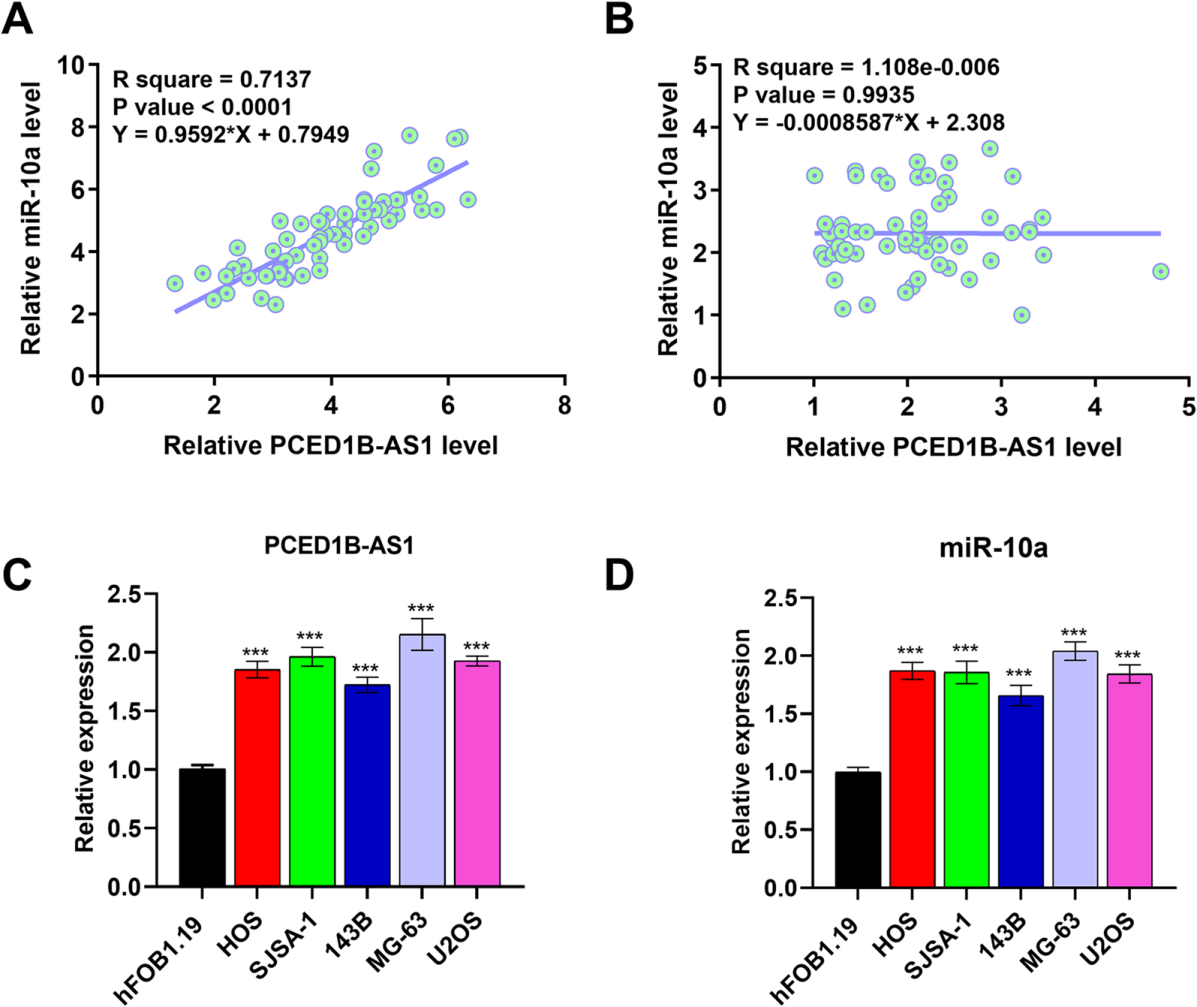
Expression levels of PCED1B-AS1 and miR-10a were positively correlated across OS tissues. The correlation of PCED1B-AS1 to miR-10a across OS tissues (**A**) and non-tumor tissues (**B**) was studied with linear regression. The expression of PCED1B-AS1 (**C**) and miR-10a (**D**) in OS cell lines were analyzed using qPCR. *p < 0.05; ***p < 0.001

Correlation analysis performed using Pearson’s correlation coefficient showed that PCED1B-AS1 expression levels were positively correlated with miR-10a expression levels across OS tissues (Fig. 2A, *p* < 0.0001), but not non-tumor tissues (Fig. 2B, *p* = 0.9935). RT-qPCR was also performed to analyze the expression of PCED1B-AS1 and miR-10a in OS cell lines and a normal cell line. As shown in Fig. 2C, D, both PCED1B-AS1 and miR-10a were upregulated in OS cells compared to the normal hFOB1.19 cell line, further confirming the upregulation of PCED1B-AS1 and miR-10a in OS.

### PCED1B-AS1 siRNA silencing downregulated miR-10a through methylation

**Fig. 3:**
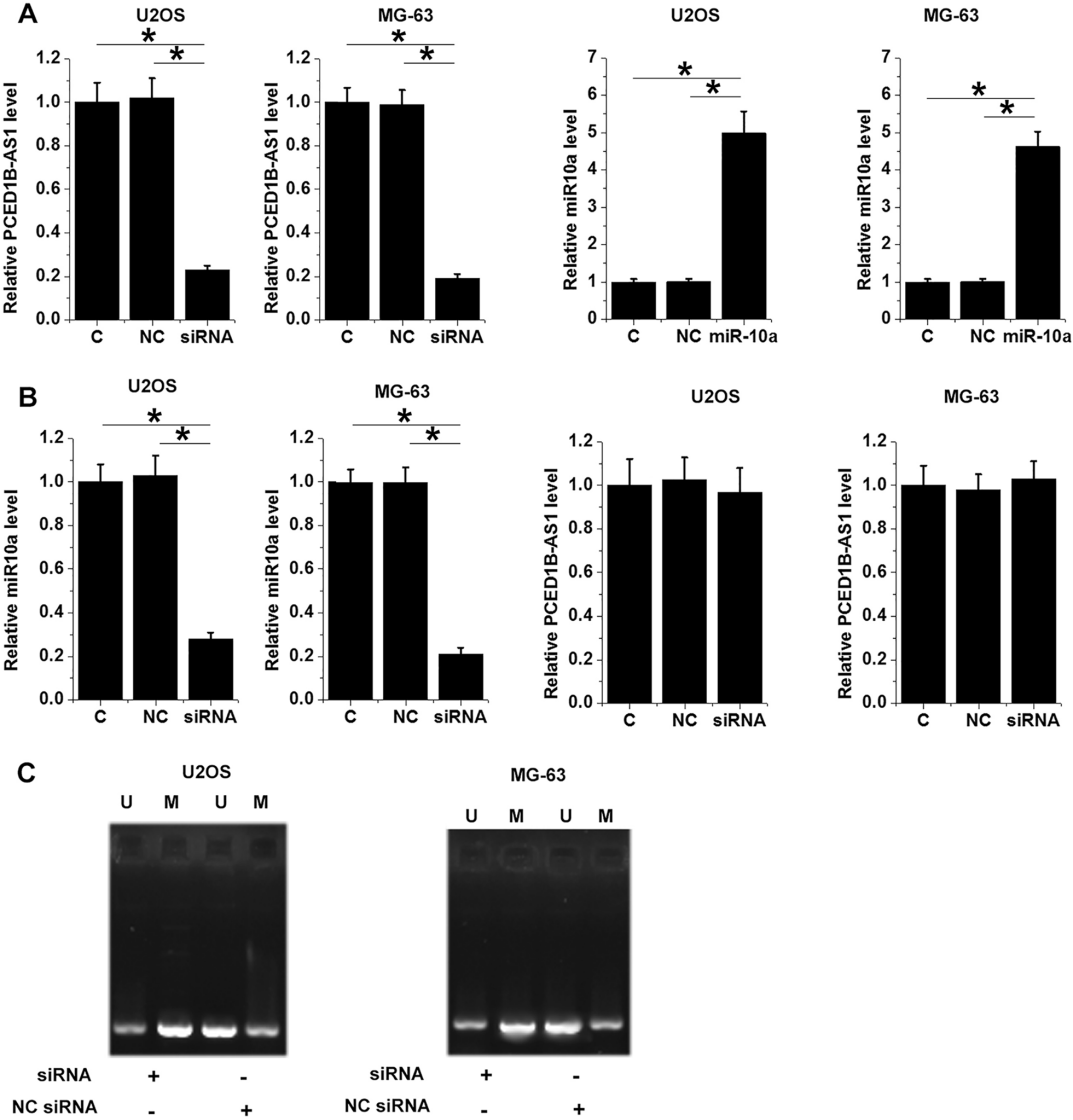
PCED1B-AS1 siRNA silencing downregulated miR-10a via methylation. PCED1B-AS1 was downregulated and miR-10a was upregulated in U2OS and MG-63 (**A**). The role of PCED1B-AS1 siRNA in miR-10a accumulation and the role of miR-10a in PCED1B-AS1 accumulation (**B**) were studied with RT-qPCR. The role of PCED1B-AS1 siRNA silencing in miR-10a gene methylation was studied with MSP (**C**). *p < 0.05

Cell transfections were performed for interaction analysis. Considering that PCED1B-AS1 has already been accumulated to high levels in OS, PCED1B-AS1 siRNA silencing was performed. At 48 h after transfection, RT-qPCR analysis showed that PCED1B-AS1 was downregulated by 4.2-fold and miR-10a was upregulated by 5.1-fold (Fig. 3A, *p* < 0.05). RT-qPCR analysis also showed that PCED1B-AS1 siRNA transfection decreased miR-10a accumulation (Fig. 3B, *p* < 0.05). In contrast, miR-10a overexpression failed to significantly affect PCED1B-AS1 expression (Fig. 3B). Therefore, PCED1B-AS1 may serve as an upstream regulator of miR-10a in OS. To explore the potential mechanism, the role of PCED1B-AS1 in regulating miR-10a promoter region methylation was analyzed by MSP. MSP analysis showed that PCED1B-AS1 siRNA silencing increased miR-10a gene methylation (Fig. 3C).

### PCED1B-AS1 siRNA silencing inhibited OS cell proliferation, invasion, and migration via miR-10a

**Fig. 4:**
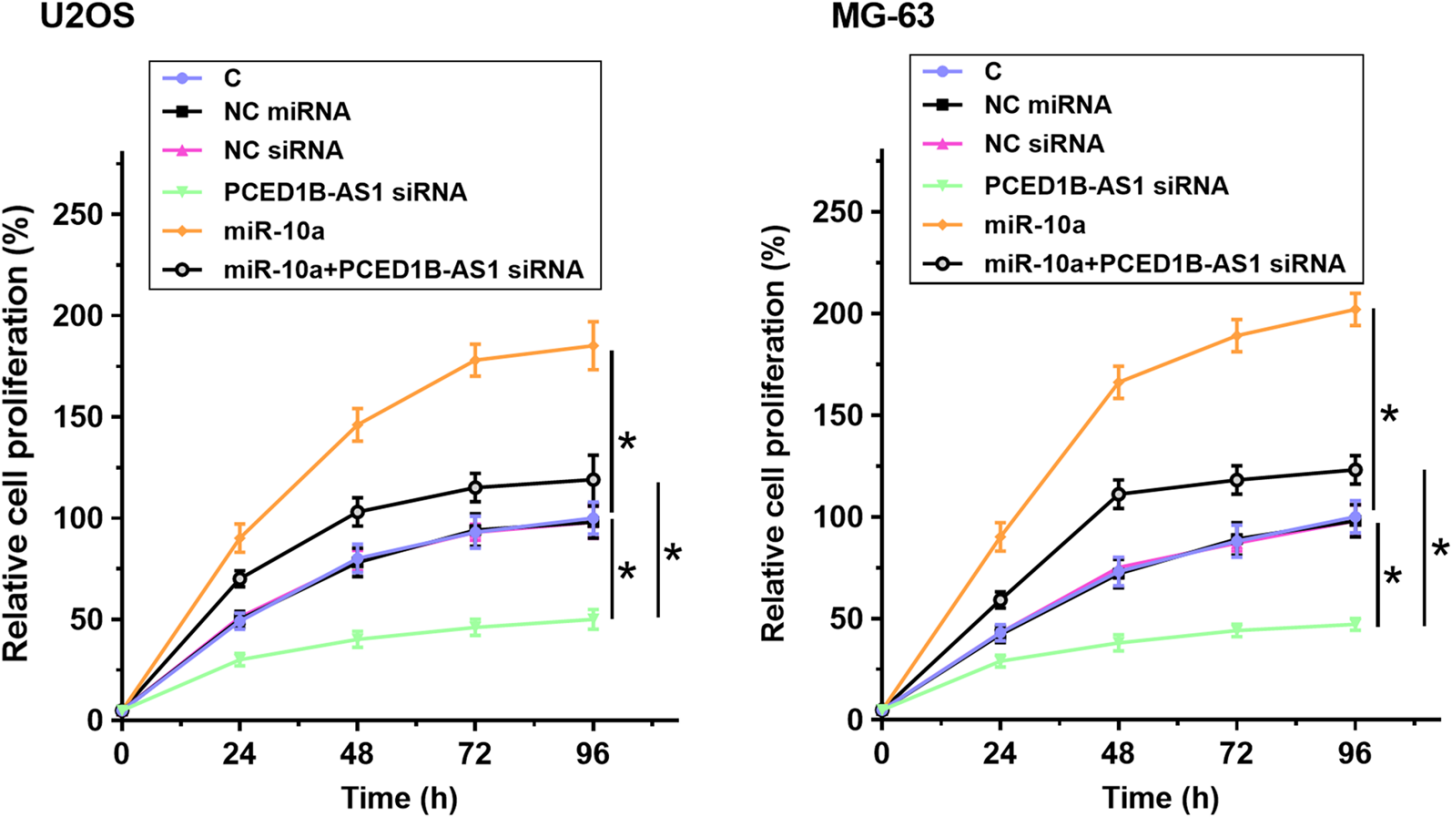
PCED1B-AS1 siRNA silencing inhibited OS cell proliferation through miR-10a. The roles of PCED1B-AS1 siRNA silencing and miR-10a overexpression in the proliferation of U2OS and MG-63 cells were analyzed by cell proliferation assay. *p < 0.05

**Fig. 5:**
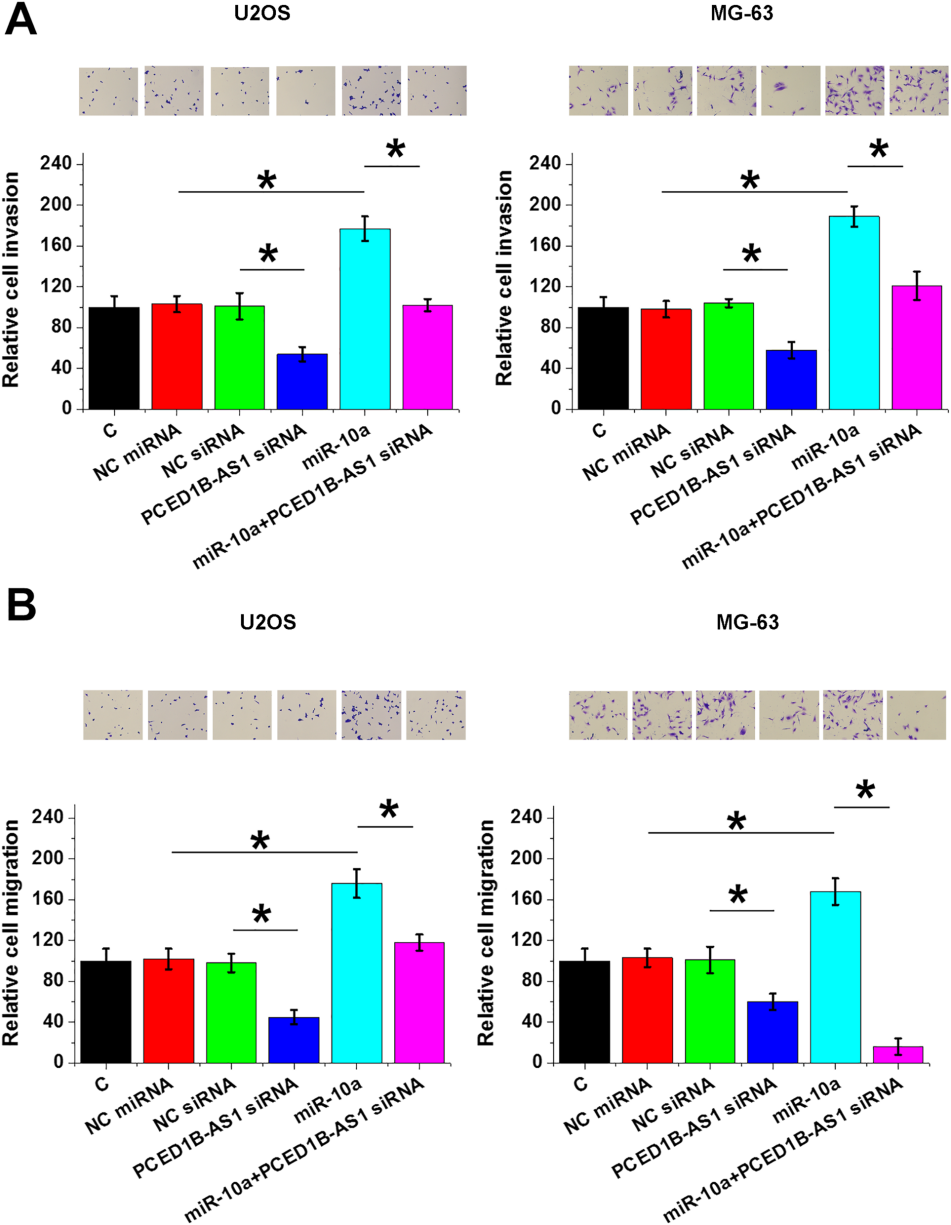
PCED1B-AS1 siRNA silencing inhibited OS cell invasion and migration through miR-10a. The effects of PCED1B-AS1 siRNA silencing and miR-10a overexpression on the invasion (**A**) and migration (**B**) of U2OS and MG-63 cells were analyzed by Transwell assay. *p < 0.05

**Fig. 6:**
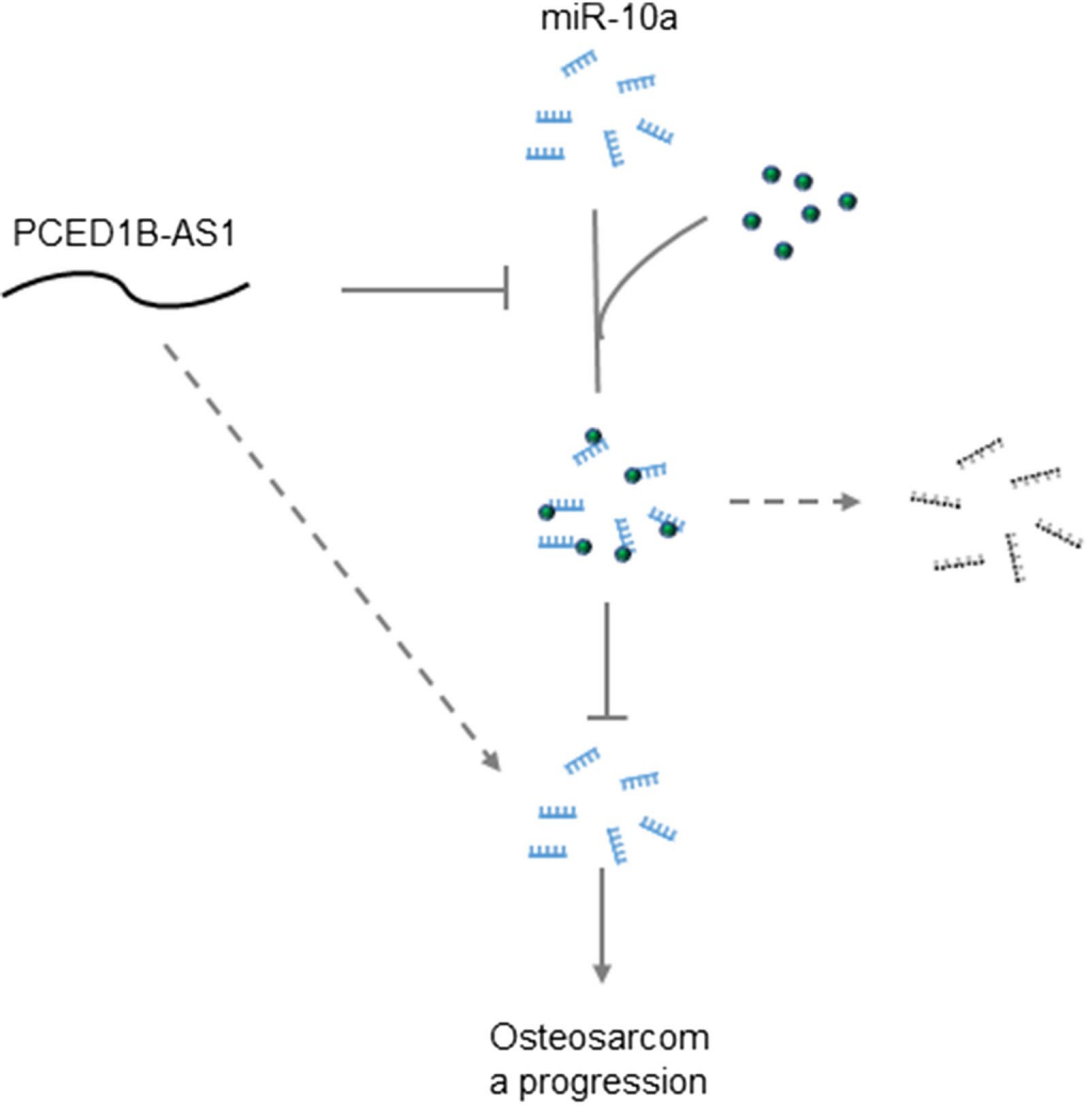
The graphical presentation of the pathway of PCEDB1-AS1 lncRNA and miR-10a through methylation. PCEDB1-AS1 inhibited miR-10a methylation, which in turn increased miR-10a expression and promoted OS progression

The roles of PCED1B-AS1 siRNA silencing and miR-10a overexpression in the proliferation, invasion, and migration of U2OS and MG-63 cells were analyzed by cell proliferation (Fig. 4), invasion (Fig. 5A), and migration (Fig. 5B) assays. Compared with control cells without transfection, PCED1B-AS1 siRNA silencing decreased cell proliferation, invasion, and migration, and miR-10a overexpression increased cell proliferation, invasion, and migration (*p* < 0.05). In addition, miR-10a overexpression reversed the inhibitory effects of PCED1B-AS1 siRNA silencing on cell proliferation, invasion, and migration (*p* < 0.05) (Fig. 6).

## Discussion

We analyzed the interactions between PCED1B-AS1 and miR-10a in OS. PCED1B-AS1 and miR-10a were both upregulated in OS, and PCED1B-AS1 siRNA silencing decreased miR-10a methylation to suppress cell proliferation.

It has been reported that PCED1B-AS1 plays a critical role in macrophage apoptosis and autophagy [18]. However, its functions in cancers have only been investigated in glioma [14]. PCED1B-AS1 is accumulated to high levels in glioma and regulates PCED1B through miR-194-5p to promote glioma [14]. Moreover, PCED1B-AS1 overexpression has been found to promote pancreatic ductal adenocarcinoma [15] and hepatocellular carcinoma [16]. We reported PCED1B-AS1 upregulation in OS. In addition, PCED1B-AS1 siRNA silencing reduced proliferation and movement of OS cells. Therefore, PCED1B-AS1 might play an oncogenic role in OS, and PCED1B-AS1 inhibition might serve as a potential target for the treatment of OS. However, clinical trials and animal model studies are needed to analyze the in vivo function of PCED1B-AS1 in OS and explore its potential clinical values.

Different roles of miR-10a have been reported in different types of cancers [17, 19]. For instance, miR-10a was overexpressed in oral squamous cell carcinoma and promotes glucose metabolism in cancer cells by regulating glucose transporter 1 the expression [19]. In contrast, miR-10a is downregulated in colorectal cancer and suppresses epithelial-to-mesenchymal transition [19]. It has been reported that miR-10a is upregulated in OS [20], while its functions in OS remain unclear. Consistently, our study observed miR-10a upregulation in OS and its enhancing effects on OS cell proliferation, invasion, and migration. Therefore, miR-10a might play an oncogenic role in OS by promoting cancer cell proliferation.

Glaich et al. reported that DNA methylation directly affects miRNA biogenesis. It is unknown whether lncRNA could regulate miRNA methylation. The key finding of the present study is that PCED1B-AS1 silencing downregulates miR-10a via methylation. However, methylation factors involved in this process remain to be further analyzed. Previous studies have shown that lncRNAs may interact with DNA methyltransferase [21]. For instance, HOTAIR upregulates DNA methyltransferases in hepatocellular carcinoma to epigenetically suppressed miR-122 [21]. In another study, PVT1 could recruit DNMT1 through EZH2 to miR-18b-5p gene promoter, thereby suppressing gene expression through methylation [22]. Future studies may focus on the potential interaction between PCED1B-AS1 and these methylation factors. It is unknown whether PCED1B-AS1 directly interacts with methylation factors to regulate miR-10a RNA gene via methylation or other mediators. In addition, we only observed the positive correlation between PCED1B-AS1 and miR-10a across OS tissue samples, but not non-tumor tissue samples. Therefore, the interaction between PCED1B-AS1 and miR-10a is likely mediated by certain pathological factors.

Our data illustrated that PCED1B-AS1 silencing is likely a promising target to treat OS by negatively regulating multiple cancer cell behaviors. However, this study failed to analyze the diagnostic and prognostic values of PCED1B-AS1 for OS, especially its potential role in the early diagnosis of OS. Moreover, no in vivo experiment was performed to validate the interaction between PCED1B-AS1 and miR-10a. Future studies are still needed. With the increased understanding of the roles of non-coding RNAs in musculoskeletal conditions, novel diagnostic biomarkers and therapeutic approaches are expected to be developed [23–26].

## Conclusions

PCED1B-AS1 and miR-10a are both upregulated in OS. PCED1B-AS1 siRNA silencing might serve as a potential target for the treatment of OS by suppressing OS cell proliferation. The function of PCED1B-AS1 in OS is likely mediated by regulating miR-10a through methylation.

## Supplementary Information

**Additional file 1.** **Supplemental Table 1:** clinicopathologic characteristics of patient samples in OS. **Supplemental Table 2:** Correlation between PCEDB1-AS1 expression and clinicopathologic features in OS patients.

## Data Availability

The data were available from corresponding author upon reasonable request.
