# Supplementary material for: LncRNA PCED1B-AS1 knockdown inhibits osteosarcoma via methylation-mediated miR-10a downregulation
Source: J Orthop Surg Res. 2022 Oct 23;17:464. doi: 10.1186/s13018-022-03284-1 (PMC9590122; doi:10.1186/s13018-022-03284-1)
Supplement: Supplementary file 1 — Additional file 1. Supplemental Table 1: clinicopathologic characteristics of patient samples in OS. Supplemental Table 2: Correlation between PCEDB1-AS1 expression and clinicopathologic features in OS patients. [file 13018_2022_3284_MOESM1_ESM.docx]

**Supplemental Table 1: Associations between patients’ clinicopathologic characteristics and PCED1B-AS1 or miR-10a expression**

| **Characteristics** | **Number of cases** | **PCED1B-AS1** | | **p** | **MiR-10a** | | **p** |
| --- | --- | --- | --- | --- | --- | --- | --- |
|  |  | **High** | **Low** |  | **High** | **Low** |  |
| **Age (median = 23.2)** |  |  |  |  |  |  |  |
| ≤23.2 | 30 | 14 | 16 | >0.05 | 17 | 13 | >0.05 |
| ＞23.2 | 30 | 16 | 14 |  | 13 | 17 |  |
| **Gender** |  |  |  |  |  |  |  |
| Male | 38 | 20 | 18 | >0.05 | 17 | 21 | >0.05 |
| Female | 22 | 10 | 12 |  | 13 | 9 |  |
| **Primary tumor site** |  |  |  |  |  |  |  |
| Femur | 36 | 17 | 19 | >0.05 | 16 | 20 | >0.05 |
| Tibia | 15 | 9 | 6 |  | 10 | 5 |  |
| Other | 9 | 4 | 5 |  | 4 | 5 |  |
| **Tumor size (cm)** |  |  |  |  |  |  |  |
| ≤5 | 31 | 10 | 21 | 0.004 | 11 | 20 | 0.02 |
| ＞5 | 29 | 20 | 9 |  | 19 | 10 |  |
| **Differentiation** |  |  |  |  |  |  |  |
| Well+moderate | 37 | 20 | 17 | >0.05 | 19 | 18 | >0.05 |
| Poor+undifferentiated | 23 | 10 | 13 |  | 11 | 12 |  |
| **Enneking staging** |  |  |  |  |  |  |  |
| I | 18 | 4 | 14 | 0.013 | 4 | 14 | 0.013 |
| II | 34 | 20 | 14 |  | 20 | 14 |  |
| III | 8 | 6 | 2 |  | 6 | 2 |  |
| **TNM** |  |  |  |  |  |  |  |
| I+II | 27 | 8 | 19 | 0.004 | 7 | 20 | 0.007 |
| III+IV | 33 | 22 | 11 |  | 23 | 10 |  |
| **Distant metastasis** |  |  |  |  |  |  |  |
| Negative | 37 | 10 | 27 | <0.001 | 11 | 26 | <0.001 |
| Positive | 23 | 20 | 3 |  | 19 | 4 |  |
